# Conformation and Aggregation of Human Serum Albumin in the Presence of Green Tea Polyphenol (EGCg) and/or Palmitic Acid

**DOI:** 10.3390/biom9110705

**Published:** 2019-11-05

**Authors:** Xiaowei Sun, Haley N. Ferguson, Ann E. Hagerman

**Affiliations:** Department of Chemistry & Biochemistry, Miami University, Oxford, OH 45056, USA; sun.2766@osu.edu (X.S.); fergush2@miamioh.edu (H.N.F.)

**Keywords:** human serum albumin (HSA), fatty acid, polyphenol, protein conformation, protein aggregation, Förster resonance energy transfer (FRET)

## Abstract

Polyphenols such as epigallocatechin gallate (EGCg) may have roles in preventing some chronic diseases when they are ingested as components of plant-based foods and beverages. Human serum albumin (HSA) is a multi-domain protein that binds various ligands and aids in their transport, distribution, and metabolism in the circulatory system. In the present study, the HSA-EGCg interaction in the absence or presence of fatty acid has been investigated. Förster resonance energy transfer (FRET) was used to determine inter- and intra-domain distances in the protein with and without EGCg and palmitic acid (PA). By labeling Cys-34 with 7-(diethyl amino)-4-methylcoumarin 3-maleimide (CPM), the distance between Trp-214 at domain IIA and CPM-Cys-34 at domain IA could be established. A small amount of PA decreased the distance, while a large amount increased the distance up to 5.4 Å. EGCg increased the inter-domain distance in HSA and HSA-PA up to 2.8 and 7.6 Å, respectively. We concluded that PA affects protein conformation more significantly compared to EGCg. Circular dichroism (CD) established that EGCg affects protein secondary structure more significantly than PA. PA had little effect on the α-helix content of HSA, while EGCg decreased the α-helix content in a dose-dependent fashion. Moreover, EGCg decreased α-helix content in HSA and HSA-PA to the same level. Dynamic light scattering (DLS) data revealed that both PA and EGCg increased HSA aggregation. EGCg increased HSA aggregation more significantly and promoted formation of aggregates that were more heterogenous. Any of these effects could impact the ability of serum albumin to transport and stabilize ligands including EGCg and other polyphenols.

## 1. Introduction

**Figure 1:**
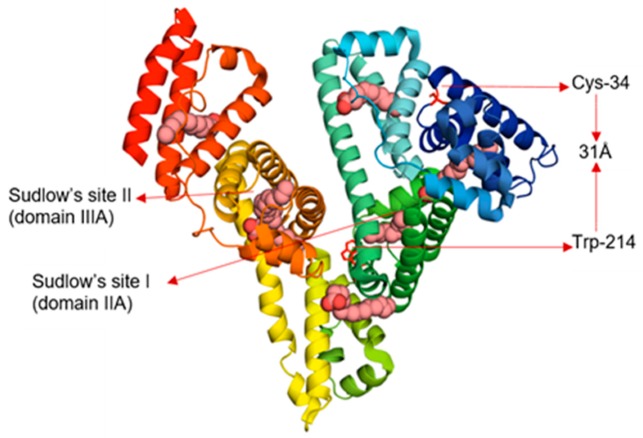
Human serum albumin (HSA) complexed with seven molecules of palmitic acid (PDB 1e7h). The different colors in HSA represent the six subdomains and the pink space filling models represent palmitic acid (PA). The Förster resonance energy transfer (FRET) distance between Trp-214 and Cys-34 in HSA is about 31 Å.

Human serum albumin (HSA) is the most abundant protein in human plasma with typical concentrations around 0.7 mM [1]. Serum albumin binds ligands such as fatty acids, glucose, drugs, excess copper, and hormones and aids in their transport, distribution, and metabolism. HSA comprises 585 amino acids, arranged mainly in alpha helices and stabilized by 17 disulfide bonds, leaving one free cysteine (Cys-34) [2,3]. The native protein has three structurally similar domains, and each domain can be divided into two subdomains, A and B. The two well-known drug-binding sites, Sudlow’s sites I and II, are hydrophobic pockets in subdomains IIA and IIIA, respectively (Figure 1) [2,4]. The single Trp residue is in subdomain IIA. HSA in solution includes 60–70% monomers with the remainder forming dimers or larger oligomers [5]. Addition of ligands such as drugs and peptide linkers can promote protein dimerization and ultimately aggregation through cross-linking [6].

HSA is an allosteric protein that can undergo conformational changes in response to many factors including pH change and ligand binding [1,7,8]. For example, adding long chain saturated fatty acids such as octanoic acid or palmitic acid (PA) to HSA can introduce protein conformational changes [9,10]. Fatty acids bind to HSA in at least seven different binding sites involving all six subdomains of the protein, including Sudlow’s sites I and II, with particularly high affinity for the latter (Figure 1) [9,10]. Allosteric changes induced by fatty acid binding alter the ability of HSA to transport other metabolites and drugs including Mn(III)-heme [11], the hormone thyroxine [12], insulin [13], and many sulfonylurea drugs [14].

**Figure 2:**
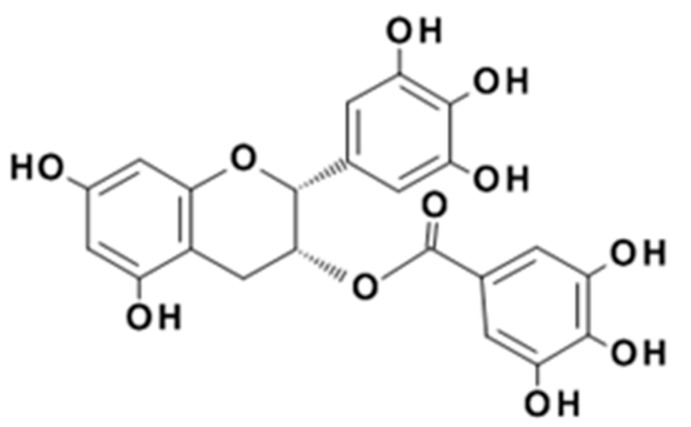
The chemical structure of the bioactive compound, (-)-epigallocatechin gallate (EGCg).

Polyphenols are plant natural products comprising more than one aromatic ring and more than one phenolic functional group [15]. The characteristic phenolic reactivity is particularly potent for the high molecular weight polyphenols commonly called tannins. These compounds are powerful antioxidants [16], excellent metal chelators [17], and not only bind protein but also induce protein precipitation under favorable conditions [18]. It is widely reported that polyphenols such as epigallocatechin gallate (EGCg), the major polyphenolic compound found in green tea (Figure 2) [19], may have roles in preventing some chronic diseases when ingested as components of plant-based foods and beverages [20]. Although EGCg is a weak protein precipitating agent compared to higher molecular weight polyphenols, its potential to modify protein solubility could be related to its putative role in protein aggregation diseases such as Alzheimer’s disease [21].

After ingestion, EGCg has limited stability in the gastrointestinal tract [22] but some of the material reaches the circulatory system where it is stabilized and carried by HSA [23]. An in vitro study has demonstrated that 1 mM EGCg is fully bound to 1.5 mM HSA within 5 min [23]. Fluorescence studies have shown that the binding constant (Kd) for EGCg and fatty acid-free HSA is about 14 μM [24,25]. Docking simulations and competitive binding experiments suggest that EGCg binds specifically to a hydrophobic pocket on HSA with more significant overlap of the nearby Sudlow’s site I and less interaction with Sudlow’s site II [26,27,28,29,30]. Recently, studies using isothermal titration calorimetry revealed that additional EGCg binds to the HSA surface non-specifically at multiple binding locations with very low affinity, with estimated Kd values in the millimolar range [27]. Upon binding, EGCg induces conformational changes in HSA and decreases its α-helical content [25,26,29,31]. Based on site-specific binding accompanied by activity changes, EGCg has been proposed as an allosteric effector for some proteins [32].

The one-on-one interaction between EGCg and serum albumin has been thoroughly examined [24,25,26,27,28,29,30,31]. However, under physiological conditions, serum albumin is typically loaded with one or more ligands consistent with its role as a carrier protein [1]. In vivo, EGCg would rarely encounter apo-serum albumin, but instead would bind the protein cooperatively or competitively with ligands such as fatty acids, metals, or drugs. To the best of our knowledge, nothing is known about how the endogenous ligands such as fatty acids or trace metals affect EGCg-serum albumin binding. A few studies have demonstrated that EGCg competes with 5-fluorouracil and related drugs for binding sites on serum albumin [30,33]. However, EGCg, resveratrol, and retinol simultaneously bind serum albumin at unique binding sites with minimal structural or chemical cross-talk between the ligands [34]. A knowledge gap that limits exploitation of the pharmaceutical potential of EGCg is poor understanding of how natural endogenous ligands of serum albumin combined with exogenous ligands such as EGCg affect the protein.

We hypothesized that EGCg may induce conformational changes not only in free HSA but also in the HSA-fatty acid complexes typically found in vivo. Furthermore, we proposed that EGCg would aggregate HSA-fatty acid complexes more efficiently than it aggregates the apoprotein, due to increased hydrophobicity of the HSA-fatty acid complex. We used several spectroscopic techniques including FRET (Förster resonance energy transfer), CD (circular dichroism), and DLS (dynamic light scattering) to study the details of the protein–polyphenol interaction in the presence and absence of the long chain saturated fatty acid PA. We suggest that this study will facilitate better understanding of the transport of the common dietary component and nutraceutical, EGCg, in human plasma as well as provide insights into potential interactions between EGCg and metabolites or drugs that are transported by serum albumin [11,12,13,14,30,35].

## 2. Materials and Methods 

### 2.1. Materials

PA was purchased from Ultra Scientific (Philadelphia, PA 19122, USA), while fatty acid-free albumin from human serum was from Sigma-Aldrich (St. Louis, MO 63103, USA). EGCg (>95%) was obtained from Lipton Tea Co (Secacus, NJ 07094, USA). Reagent grade sodium phosphate was used to prepare 20 mM, pH 7.0 buffer used in all experiments, and guanidine hydrochloride was purchased from Sigma-Aldrich. HSA was labeled with one of two fluorescent probes, 6-propionyl-2-(dimethylamino) naphthalene (prodan) or 7-(diethyl amino)-4-methylcoumarin 3-maleimide (CPM), for FRET distance determination. Prodan was purchased from Life Technologies (Waltham, MA 02451, USA), while CPM was obtained from Chemodex (Buckingham, Bucks MK18 1EG, UK).

### 2.2. HSA Labeled with CPM

To label HSA with CPM, 10 µM of the protein was prepared in phosphate buffer and incubated with 6 M guanidine hydrochloride at 25 °C for 4 h [36]. CPM (150 μM) was prepared in 50% aqueous dimethyl sulfoxide and added to the solution to achieve 15 molar equivalents of HSA. The reaction was stopped after 4 h by adding 0.5 μL of 14 M β-mercaptoethanol [37]. The excess reagents and guanidine hydrochloride were removed by dialysis for 24 h at 4 °C against phosphate buffer, changing the dialysis buffer every 5 h. After dialysis, the samples were centrifuged at 17,000× *g* for 20 min to remove undissolved materials [37]. The Bradford assay [38] was used to determine the concentration of HSA and CPM-labeled HSA using bovine serum albumin as the standard protein. The incorporation of CPM was determined by direct spectrophotometry, using the extinction coefficient of 30,000 M^−1^ cm^−1^ at 383 nm [36]. The labeling efficiency was above 80%.

### 2.3. HSA Labeled with Prodan

Prodan was suspended in phosphate buffer and sonicated for an h to obtain a 300 μM solution [36]. The HSA-prodan complex was prepared by reacting 30 μM prodan with 1 μM HSA at 25 °C for 30 min [36]. The preparation had no fluorescence emission at 520 nm indicating there was no free prodan in the sample after the reaction was complete (Figure S1).

### 2.4. Reaction Mixtures

For FRET experiments, 1 μM HSA and HSA-CPM solutions were prepared with either PA (0 to 60 equivalents) or EGCg (0 to 25 equivalents). EGCg (0 to 25 equivalents) was also titrated into 1 μM HSA-PA and HSA-CPM-PA mixtures to assess its effect on the protein-fatty acid complex. Only HSA-prodan was titrated with different concentrations of EGCg; HSA-prodan-PA could not be used since PA replaces prodan on HSA [36]. Unlabeled HSA (5 μM) was prepared with EGCg (0 to 25 equivalents) and/or PA (0 to 60 equivalents) for CD and DLS measurements. Three independent sample replicates were prepared for FRET, CD, and DLS measurements.

PA has low solubility, which makes the preparation of HSA-PA complexes complicated. Curry’s method was modified to obtain HSA-PA complexes [10]. After adding PA (2.5 mM) to 5 mL phosphate buffer, the mixture was incubated for 15 min at 50 °C. The PA suspension was then sonicated for 30 min before mixing with solutions of labeled or unlabeled HSA. After 30 min at room temperature the samples were centrifuged at 12,000× *g* for 10 min to remove undissolved PA.

EGCg was prepared in water and the concentration was determined by UV spectrometry based on its extinction coefficient (9700 M^−1^ cm^−1^ at 280 nm). EGCg-protein complexes were prepared by mixing the EGCg with the protein and incubating for 30 min at room temperature.

### 2.5. FRET Measurement

Fluorescence and absorbance measurements were used to calculate the FRET distance between Trp-214 and the prodan binding site or between Trp-214 and Cys-34-CPM. HSA and HSA-CPM or HSA and HSA-prodan was used as control for FRET distance comparison. A PerkinElmer LS 55 fluorescence spectrometer was used to measure sample fluorescence and an Agilent 8453 UV-visible spectrometer was used to measure sample absorbance at room temperature. The excitation wavelength for measuring fluorescence was 295 nm and both slit widths were set at 5 nm. The data were collected from 310 to 600 nm. Buffer blanks containing the appropriate concentration of CPM, PA, and/or EGCg were subtracted from the fluorescence data obtained with the reaction mixtures.

The distance between the intrinsic fluorophore Trp-214 (λ_ex_ = 295 nm, λ_em_ = 340 nm) and fluorescent labels within 60 Å can be determined using FRET. Free CPM has only weak fluorescence, but protein bound CPM is strongly fluorescent (λ_ex_ = 384 nm, λ_em_ = 470 nm). Free prodan has a strong emission at 520 nm that is easily differentiated from protein bound prodan (λ_ex_ = 360 nm, λ_em_ = 445 nm). In our experiments, excitation at 295 nm yielded the expected emission at 470 or 445 nm for CPM- or prodan-labeled protein, respectively. To calculate the distance between prodan or CPM and the HSA intrinsic fluorophore Trp-214, the following Equations (1)–(4) were used:

(1) $$E=\;\frac{R_{0}^{6}}{R_{0}^{6}+R^{6}}$$

(2) $$R_{0}=0.211{\left(\kappa^{2}\phi_{d}J\eta^{-4}\right)}^{1/6}$$

(3) $$J\left(\lambda \right)=\int F_{d}\left(\lambda \right)\varepsilon_{a}\left(\lambda \right)\lambda^{4}d\lambda /\int F_{d}\left(\lambda \right)d\lambda$$

(4) $$E=1-F_{da}/F_{d}$$

According to Förster’s theory [39,40,41], the efficiency of energy transfer (*E*) depends on the distance R (Å) between donor and acceptor fluorophores. *R*_0_ is the distance between donor and acceptor when the energy transfer efficiency equals 50%. The refractive index of the medium, η, is 1.4 for this experiment. A value of 2/3 was used for $\kappa^{2},$ the orientation factor. The quantum yield of the donor in the absence of the acceptor, $\Phi_{d}$, is 0.14 for Trp. In Equation (3), $F_{d}\left(\lambda \right)$ is the donor fluorescent intensity at the wavelength λ and $\varepsilon_{a}\left(\lambda \right)$ is the acceptor molar extinction coefficient [36,37]. J is the spectral overlap integral between the donor emission spectrum (Trp-214) and the acceptor absorbance spectrum (prodan or CPM), and was calculated by a|e—UV-Vis-IR spectral software FluorTools [42]. Equation (4) calculates the energy transfer value (E) based on the decrease of donor fluorescence intensity. $F_{d}$ is the donor fluorescence intensity, measured in the absence of acceptor, while $F_{da}$ is the donor fluorescence intensity when the acceptor is present.

### 2.6. CD Measurements

Circular dichroism was used to assess the secondary structure of HSA in the presence of EGCg and/or PA. A CD spectrometer MODEL 435 from AVIV biomedical, Inc. was used to measure the spectra at 25 °C in a quartz cuvette with 1 mm path length. The bandwidth was set at 1 nm, the speed was set at 50 nm min^−1^, and the data were collected from 260 to 190 nm. Each spectrum was the average of three scans and three independent replicates of each sample were analyzed. For each sample, a background spectrum obtained with the appropriate concentration of EGCg was subtracted. To calculate the α-helical content in HSA at different conditions, the following Equations (5) and (6) were used [43].

(5) $$\mathrm{MRE}=\;\frac{\mathrm{observed}\;\mathrm{CD}}{C_{p}\times n\times l\times 10}$$

(6) $$\alpha -\mathrm{helix}\left(\%\right)\;=\;\frac{\left(-{\mathrm{MRE}}_{208}-4000\right)}{33000-4000}\times 100$$

Using Equation (5), the mean residual ellipticity (MRE) can be calculated. $C_{p}$ is the HSA molar concentration, n is the number of amino acid residues in HSA, and l mm is the path length. The α-helical content in protein was calculated using Equation (6). ${\mathrm{MRE}}_{208}$ is the calculated MRE value at 208 nm, 4000 is the MRE value of β-form and random coil conformations at 208 nm, and 33,000 is the MRE value of α-helices at 208 nm.

### 2.7. DLS Measurements

A Malvern dynamic light scattering system (DLS) was used to measure protein aggregation at 25 °C in a 10 mm disposable micro cuvette. All samples were equilibrated in the cuvette for 2 min, and then scanned 15 times. Three independent sample replicates were measured to check repeatability. The data output includes information about particle diameter, reported as size distribution, and relative quantity in solution, reported as intensity, for each peak.

## 3. Results

### 3.1. Interdomain Distances

**Figure 3:**
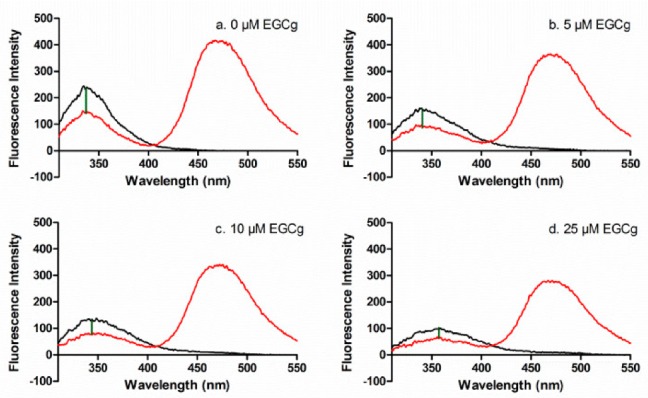
Fluorescence emission spectra for 1 μM HSA (black) and HSA-CPM (red) with 0 μM (**a**), 5 μM (**b**), 10 μM (**c**), 25 μM (**d**) EGCg. The difference in intensity at 340 nm (Δ_em_) between HSA and HSA-CPM (green line) indicates the energy transfer.

FRET was used to determine the inter-domain distance between CPM at the single sulfhydryl group Cys-34 (subdomain IA) and Trp-214 (subdomain IIA) in apoprotein and in the presence of EGCg and/or PA. The fluorophore Trp is excited at 295 nm, and the emission of Trp at 340 nm can excite CPM that is within 60 Å of the Trp residue, with reduction in the Trp emission inversely proportional to the distance between the Trp and CPM (Δ_em_, Figure 3a).

EGCg complicated the FRET analysis because EGCg quenches the intrinsic fluorescence of HSA by binding to the protein near the Trp residue [26]. EGCg quenched HSA as expected (black lines, Figure 3a–d) and also quenched CPM-HSA (red lines, Figure 3a–d). In addition, EGCg induces a small red shift in the HSA or CPM-HSA emission spectrum (red lines, Figure 3a–d), probably because EGCg binds to HSA at the hydrophobic pocket and disrupts a salt bridge [44]. Since the EGCg-induced spectral changes applied to both HSA and HSA-CPM, they did not affect the calculated FRET energy transfer from Trp-214 to the CPM label. The data showed that EGCg diminished the Δ_em_, indicating that the distance between Trp and CPM increased as more EGCg was added (Figure 3a–d). EGCg also quenched the Trp fluorescence of HSA-PA and HSA-CPM-PA, and decreased Δ_em_ in the PA-loaded protein (Figures S2–S4). The overlap of the HSA donor emission spectrum and the HSA-CPM acceptor absorption spectrum is suitable for J value calculation (Figure S5, Table S1).

In the apoprotein, the mean distance between Trp-214 and CPM-Cys-34 was 30.2 ± 1.5 Å, consistent with previous reports [36]. For each treatment, the change in distance between Trp-214 and CPM-Cys-34 was calculated by subtracting the distance determined in the presence of the ligand from the distance determined for apoHSA. A negative change in distance indicated that the ligand moved protein domains closer together, while a positive change in distance corresponded to increased distance between the labeled positions.

**Figure 4:**
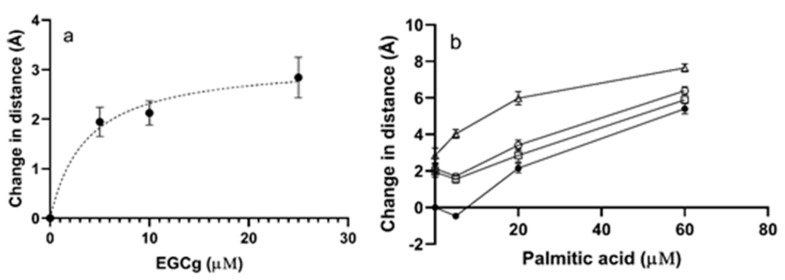
FRET distance between Trp-214 and CPM for HSA (1 μM with 0–25 μM of EGCg and/or 0–60 μM PA. The data points are means calculated from three replicates and the error bars indicate SEM. (**a**) FRET distance between Trp-214 and CPM with different concentrations of EGCg (0–25 μM). Bmax was 3.2 Å and apparent EC_50_ was 3.7 μM based on a saturation binding model for one ligand. (**b**) FRET distance between Trp-214 and CPM in the presence of PA (5, 20, 60 μM) and EGCg (0, 5, 10, 25 μM). ●, 0 μM EGCg; □, 5 μM EGCg; ○, 10 μM EGCg; Δ, 25 μM EGCg. The saturation binding model is not appropriate for PA binding to HSA.

EGCg increased the distance between Trp-214 and CPM-Cys-34 in the absence of PA (Figure 4a, Table S2). The maximum increase detected was 2.8 ± 0.7 Å at a molar ratio of 25 EGCg to 1 HSA (Figure 4a). The effect was dose dependent and fit a one binding site isotherm, with a calculated maximum change in distance of 3.2 Å and an apparent EC_50_ of 3.7 μM. The effect of PA on the protein was more complex (Figure 4b, Table S2). A small amount of PA added to the protein decreased the distance between Trp-214 and CPM-Cys-34 slightly, indicating that fatty acid at a low level made the structure somewhat more closed. Excess PA relaxed the protein, increasing the distance between the domains. The maximum decrease in distance induced by PA was 0.5 +/− 0.1 Å, while the maximum increase was 5.4 +/− 0.5 Å (Figure 4b). Palmitic acid induced larger changes in the protein structure than EGCg.

When EGCg was added to the HSA-PA complexes, the distance between the domains generally increased but the magnitude of the increase was dependent on the amount of PA (Figure 4b, Table S2). The EGCg-induced increase in the Trp-to-Cys distance in HSA treated with 5 μM PA was less than the EGCg-induced increase for apoprotein, consistent with the ability of small amounts of PA to close the protein structure. The EGCg-induced increase in distance in HSA with excess PA was larger than the increase for apoprotein. The data suggested that the effects of the ligands were close to additive. For example, the distance increase was 7.6 +/− 0.4 Å for protein treated by the highest concentrations of PA and EGCg compared to 8.2 Å predicted for an additive effect.

FRET was used to determine the intra-domain flexibility of domain II by measuring energy transfer from Trp-214 to prodan, which binds to Sudlow’s site I [36,45]. The method could only be used to examine the effects of EGCg because Sudlow’s site I binds fatty acids, so addition of PA diplaces the prodan label [36]. Although the EGCg binding site overlaps Sudlow’s site I [26], addition of EGCg to prodan-labeled protein did not increase the fluorescence characteristic of free prodan (520 nm) (Figure S1), indicating that both ligands could simultaneously bind to the protein. The distance between prodan and Trp-214 in HSA was 25 ± 0.7 Å. Addition of EGCg did not change the distance (data not shown).

### 3.2. Secondary Structure

**Figure 5:**
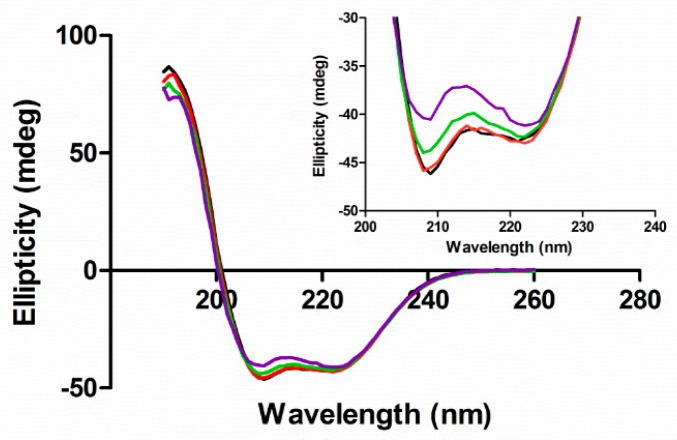
Circular dichroism (CD) spectra of HSA (5 μM) in the absence and presence of EGCg in 20 mM phosphate buffer at pH 7. The concentrations of EGCg were 0 μM (black), 25 μM (red), 50 μM (green), and 125 μM (purple).

**Figure 6:**
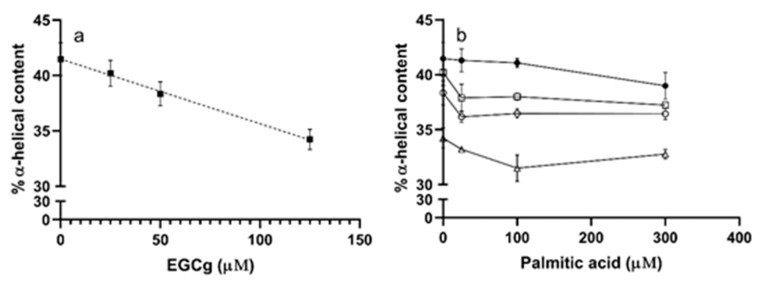
α-helical content in HSA (5 μM) with 0–125 μM of EGCg and/or 0–300 μM PA. Each point was calculated from three replicates with SEM for error bars. (**a**) HSA (5 μM) α-helical content with different concentrations of EGCg (0–125 μM). The line was linearly fit (Prism) to yield slope—0.29 and r^2^ = 0.72. (**b**) HSA (5 μM) α-helical content in the presence of PA (0, 25, 100, 300 μM) and EGCg (0, 25, 50, 125 μM). ●, 0 μM EGCg; □, 25 μM EGCg; ○, 50 μM EGCg; Δ, 125 μM EGCg.

We used circular dichroism to assess the secondary structure of HSA in the presence of different ligands by monitoring the π-π* and n-π* transitions of peptide bonds in α-helices [46]. The CD spectra of HSA had negative bands at 209 and 222 nm (Figure 5 black line). When EGCg was added to HSA, the intensity of both bands decreased without any shift in the band maxima (Figure 5 red, green, and purple lines). The intensity change was larger in the band at 209 nm than that at 222 nm. EGCg decreased the α-helix content of HSA a small amount with a somewhat linear dose dependence (R^2^ = 0.73) with about 7% less α-helix at the highest EGCg concentration compared to the apoprotein (Figure 6a, Table S3).

PA had little effect on the α-helix content of HSA (Figure 6b, Table S3), with the CD spectra in the presence of ligand similar to the spectrum of apoHSA (Figures S6–S8). The effect of EGCg on PA-treated protein is similar to the effect on apoprotein, with decreasing α-helix content proportional to the amount of EGCg added (Figure 6b, Table S3). Overall, EGCg induced a loss of the stability of α-helix, and affected protein secondary structure more significantly compared to PA.

### 3.3. Protein Size and Aggregation

**Figure 7:**
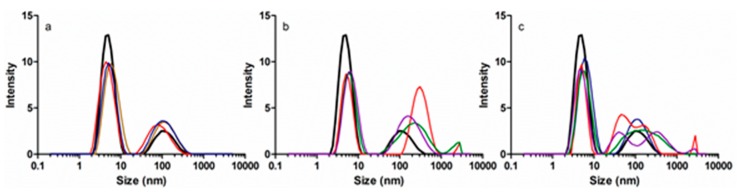
Dynamic light scattering (DLS) size distribution of (**a**) 5 μM HSA titrated with 0 μM (black), 25 μM (yellow), 100 μM (red), or 300 μM (blue) PA; (**b**) 5 μM HSA titrated with 0 μM (black), 25 μM (red), 50 μM (green), or 125 μM (purple) EGCg; (**c**) 5 μM HSA (black) and HSA (5 μM)-PA (300 μM) titrated with 0 μM (blue), 25 μM (red), 50 μM (green), or 125 μM (purple) EGCg.

Dynamic light scattering was used to monitor the changes in HSA size and aggregation upon interaction with EGCg and/or PA. Addition of PA did not change the size of the 5.3 nm HSA monomer (Figure 7a), but addition of EGCg to HSA or PA-HSA moved the monomer peak slightly to the right (Figure 7b,c) indicating an approximately 0.4 nm increase in average diameter. However, the effects of EGCg were not very consistent. The conformational changes due to EGCg are small, and DLS measurements are dynamic, so it was reasonable that protein monomer size changes were not detected in every sample.

DLS is more sensitive to protein aggregation and polydispersity than to small conformational changes. HSA solutions always contain some aggregated material, but EGCg or PA changed the area, position, and number of peaks of HSA aggregates. The effects of EGCg were not very reproducible within instrumental replicates or between chemical replicates, suggesting that the polyphenol induced heterogeneity. Generally, EGCg promoted aggregation more than PA. For apoHSA, about 23% of the total peak area was attributed to aggregates. PA increased the proportion of aggregates to about 35% (Figure 7a). EGCg increased the proportion of aggregates to about 45% for both apo HSA and PA-treated HSA (Figure 7b,c). Moreover, EGCg introduced more heterogeneity to HSA and HSA-PA based on the area, position, and number of different peaks representing aggregated protein (Figure 7b,c), while the aggregates induced by PA were more uniform (Figure 7a).

## 4. Discussion

At low concentrations the nonpolar polyphenol EGCg selectively binds a hydrophobic crevice in HSA with substantial overlap of Sudlow’s site I [26,27,28,29,30]. However, EGCg also has polar characteristics (phenolic hydroxyl groups), and at sufficiently high concentration it can bind nonspecifically to the protein surface [27]. It has been reported that 25 equivalents of EGCg are required to fill all EGCg binding sites on HSA [27,31]. We used up to 25 equivalents of EGCg to study the maximal conformational changes that could be induced by EGCg to the apoprotein and the protein loaded with PA.

Our FRET results indicated that when EGCg binds the apoprotein, it increases the HSA inter-domain distance between the Trp-214 in subdomain IIA and CPM-Cys-34 in subdomain IA. EGCg did not change the organization of structural elements within domain II, monitored with prodan. The effect of EGCg was dose-dependent and saturable with a maximum change of 2.8 Å. The effect of EGCg on inter-domain distance was similar to but smaller than the effects of pH or denaturants on serum albumin [37,41]. The maximum increase of distance between domain I and domain II induced by increasing or decreasing pH is about 10 Å [37,41], while guanidine hydrochloride increases the inter-domain distance about 11 Å [37]. The data suggest that EGCg binding does not fully denature the protein, but disrupts interdomain interactions by altering the polar environment near the hydrophobic pocket and Sudlow’s site I, thought to be a conformationally adaptable region of the protein [47,48]. The saturable nature of the EGCg effect supports the idea that this ligand modulates inter-domain distances by specific binding at the hydrophobic pocket and Sudlow’s site I. Even though EGCg at high concentration could nonspecifically bind to HSA surface, such nonspecific binding may not significantly affect protein conformation.

The effects of PA on inter-domain distances are more complex, with low levels of PA decreasing the distance between subdomains IA and IIA, and saturating levels of PA increasing the distance. In our experiment, up to 60 equivalents of PA were added to HSA to ensure that all sites are occupied [9,36]. The variable allosteric effects of PA on protein structure are a consequence of the protein’s ability to bind fatty acid at several widely distributed sites on the protein [9,10], such as FA4 and FA5 in domain III [49,50] and FA2 in subdomain IA [51], with different effects on structure as more sites are filled. Previous studies have confirmed a dose dependent effect of fatty acids on serum albumin structure, with the structure closing at low PA concentrations due to rotation of the subdomain IB-IIA inter-domain helix [52]. At higher PA concentrations the subdomain IIB-IIIA inter-domain helix tilts slightly, resulting in rotation of domains I and III away from domain II and opening up the central crevice about 10 Å [9,52,53]. These changes are consistent with our FRET data that shows that 60 equivalents of PA increases HSA inter-domain distance between domain IA and IIA up to 5.4 Å.

When added to the PA-serum albumin complex, EGCg opens the protein structure more than when the fatty acid is not present. It has been reported that Sudlow’s site I ligand binding is enhanced in the protein-fatty acid complex [11]. The increased interdomain distance induced by EGCg may indicate that EGCg more efficiently binds PA-serum albumin compared to apoprotein.

Despite the ability of PA to alter inter-domain protein distances in HSA, the CD results suggested that PA had little effect on α-helix content of the protein. Our data are consistent with other studies that have established that the overall high α-helical character of serum albumin is slightly decreased only when very high levels of fatty acids are added to the protein [36,54]. The minor changes in helical content could be due to high levels of hydrophobic ligands [55] or could be due to protein unfolding induced by micelles [56].

Adding EGCg to HSA decreased the α-helical content in a dose-dependent fashion consistent with previous studies [24,25]. Unlike its effect on inter-domain distance, EGCg affected α-helix content in a non-saturable fashion. This suggests that the effects on helical content are due to nonspecific binding, probably at the protein surface through hydrogen bonding between phenolic hydroxyls and peptide carbonyls [27,57]. The decrease in α-helical content was similar to that observed with pH changes that disrupt hydrogen bonding and electrostatic interactions in the protein [41].

When added to the HSA-PA complex, the effects of EGCg on the protein α-helical content were similar to its effect on apoHSA. EGCg overwhelmed the small effects of PA on the protein secondary structure. PA binds at specific interior sites on the protein by hydrophobic and electrostatic interactions [9,58], while EGCg binds to a specific interior hydrophobic pocket, and nonspecifically to the surface at least in part via hydrogen bonds at the peptide backbone [26,27]. Therefore, polyphenols such as EGCg are more likely to disrupt α-helices.

The ability of EGCg to precipitate protein is more well-known than its effect on protein secondary and tertiary structure. EGCg is a less effective precipitating agent than higher molecular weight polyphenols (tannins), requiring a stoichiometric ratio of 125:1 to achieve sufficient protein coating and crosslinking to initiate precipitation [28]. Our DLS data revealed that at lower EGCg:protein ratios (5:1, 10:1, 25:1), the polyphenol stimulates HSA aggregation without precipitation. The formation of aggregates was not strongly concentration dependent and yielded highly polydisperse mixtures. Unlike the site-specific binding event by EGCg that results in changes in domain substructures in the protein, aggregation apparently takes place when excess EGCg binds nonspecifically to the surface of the protein. Binding to the surface decreases helical content and starts to denature the protein, and may also create “hot spots” of hydrophobicity or masked surface charge that promote aggregate formation [59]. Since surface binding is nonspecific, some binding events may not significantly change the protein, resulting in polydisperse mixtures that form precipitates only when the polyphenol is in significant excess over the protein so that many nonspecific surface sites are filled and cross-links can form between proteins.

PA also increased protein aggregation, consistent with previous studies [60]. Compared to EGCg, PA induced formation of more homogeneous aggregates. PA binds to HSA at specific sites on the interior of the protein, mainly through hydrophobic interactions. Loading the protein with fatty acid could cause a systematic change in hydrophobicity that could increase formation of aggregates. Ligands such as fatty acids that have site-specific binding may form aggregates with defined stoichiometries, leading to mixtures that are less polydisperse than the populations of aggregates generated by a nonspecific ligand such as EGCg.

EGCg aggregated HSA-PA complexes in a fashion similar to its behavior with apoHSA. The result suggests that binding PA at interior sites of the protein does not affect EGCg binding at the surface of the protein. EGCg is well-suited to interact with hydrophobic and hydrophilic sites on the protein, because of its dual nature as an aromatic, hydrophobic compound with numerous polar hydroxyl functional groups. Thus, it is able to aggregate either the apoprotein or the less polar PA-loaded protein, in each case forming hetero-disperse aggregates due to nonspecific surface binding on the protein. Although it has been suggested that EGCg has therapeutic potential because of its ability to remodel fibrillar proteins [61,62], our data indicate a rather nonselective effect, suggesting that the potential for EGCg to control protein fibril formation in disease states may be limited.

## 5. Conclusions

This study shows that the dietary polyphenol, EGCg, and fatty acids, such as PA, simultaneously bind to serum albumin and influence the structure of the protein. The highly hydrophobic fatty acid and the amphipathic polyphenol appear to have unique binding characteristics with the protein, leading to the conclusion that in vivo the fatty acid-loaded serum albumin probably does transport EGCg. However, the additive effect of the two ligands on protein features such as the interdomain distance between subdomain IA and subdomain IIA could have important consequences for drugs whose binding at Sudlow’s site I is allosterically controlled by fatty acids. Our study demonstrated that EGCg influences protein secondary structure while the effects of PA on HSA are related to protein conformation. Further studies of drug binding, transport, and release by HSA in the presence of both EGCg and fatty acids are critical to a better understanding of EGCg, which is not only a natural dietary component but also a popular dietary supplement.

## Supplementary Materials

The following are available online at https://www.mdpi.com/2218-273X/9/11/705/s1, Figure S1: Fluorescence spectra of HSA and HSA-prodan with different concentrations of EGCg. Figure S2: Fluorescence emission spectra for HSA (1 μM)-PA (5 μM) and HSA-CPM (1 μM)-PA (5 μM) with 0 μM (a), 5 μM (b), 10 μM (c), 25 μM (d) EGCg. Figure S3: Fluorescence emission spectra for HSA (1 μM)-PA (20 μM) and HSA-CPM (1 μM)-PA (20 μM) with 0 μM (a), 5 μM (b), 10 μM (c), 25 μM (d) EGCg. Figure S4: Fluorescence emission spectra for HSA (1 μM)-PA (60 μM) and HSA-CPM (1 μM)-PA (60 μM) with 0 μM (a), 5 μM (b), 10 μM (c), 25 μM (d) EGCg. Figure S5: Overlap of the donor emission spectrum (Trp-214) and the acceptor absorption spectrum (HAS-CPM). Figure S6: CD spectra of 5 μM HSA and 5 μM HSA in the presence of 25 μM PA, and 0–125 μM EGCg. Figure S7: CD spectra of 5 μM HSA and 5 μM HSA in the presence of 100 μM PA, and 0–125 μM EGCg. Figure S8: CD spectra of 5 μM HSA and 5 μM HSA in the presence of 300 μM PA, and 0–125 μM EGCg. Table S1: J, *R*_0_, and *R* values for HSA, HSA-PA, and HSA-PA-EGCg for three replicates. Table S2: The change in distance (Å) between Trp-214 and CPM induced by addition of EGCg and/or palmitic acid. Table S3: The α-helical content (%) of HSA with various amounts of EGCg and/or palmitic acid.
