# Supplementary material for: Conformation and Aggregation of Human Serum Albumin in the Presence of Green Tea Polyphenol (EGCg) and/or Palmitic Acid
Source: Biomolecules. 2019 Nov 5;9(11):705. doi: 10.3390/biom9110705 (PMC6920801; doi:10.3390/biom9110705)
Supplement: Supplementary file 1 [file biomolecules-09-00705-s001.pdf]

biomolecules

Supplementary materials

Conformation and aggregation of human serum albumin in the presence of green tea polyphenol (EGCg) and/or palmitic acid

Xiaowei Sun [1], Haley N. Ferguson [1] and Ann E. Hagerman [1,*]

[1] Department of Chemistry & Biochemistry, Miami University; sun.2766@osu.edu (X.S.) ; fergush2@miamioh.edu (H.N.F.)

* Correspondence: hagermae@miamioh.edu; Tel.: +001-513-529-2827

Received: September xx, 2019; Accepted: date; Published: date

Supplementary Materials: The following are available online at www.mdpi.com/xxx/s1, Figure S1: Fluorescence spectra of HSA and HSA-prodan with different concentrations of EGCg. Figure S2: Fluorescence emission spectra for HSA (1 μM)-PA (5 μM) and HSA-CPM (1 μM)-PA (5 μM) with 0 μM (a), 5 μM (b), 10 μM (c), 25 μM (d) EGCg. Figure S3: Fluorescence emission spectra for HSA (1 μM)-PA (20 μM) and HSA-CPM (1 μM)-PA (20 μM) with 0 μM (a), 5 μM (b), 10 μM (c), 25 μM (d) EGCg. Figure S4: Fluorescence emission spectra for HSA (1 μM)-PA (60 μM) and HSA-CPM (1 μM)-PA (60 μM) with 0 μM (a), 5 μM (b), 10 μM (c), 25 μM (d) EGCg. Figure S5: Overlap of the donor emission spectrum (Trp-214) and the acceptor absorption spectrum (HAS-CPM). Figure S6: CD spectra of 5 μM HSA and 5 μM HSA in the presence of 25 μM PA, and 0-125 μM EGCg. Figure S7: CD spectra of 5 μM HSA and 5 μM HSA in the presence of 100 μM PA, and 0-125 μM EGCg. Figure S8: CD spectra of 5 μM HSA and 5 μM HSA in the presence of 300 μM PA, and 0-125 μM EGCg. Table S1. J, R0 and R values for HSA, HSA-PA and HSA-PA-EGCg (n=3). Table S2: The change in distance (Å) between Trp-214 and CPM induced by addition of EGCg and/or palmitic acid. Table S3: The α-helical content (%) of HSA with various amounts of EGCg and/or palmitic acid.

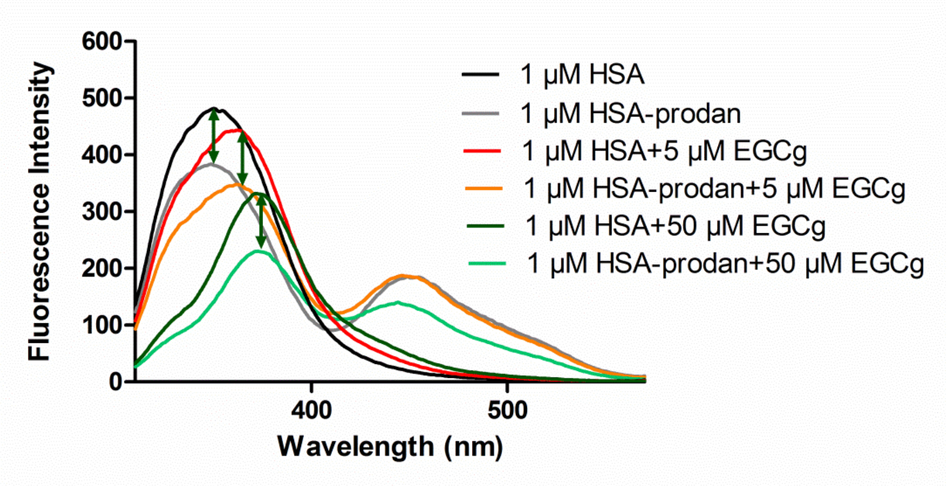

Figure S1. Fluorescence spectra of HSA and HSA-prodan with different concentrations of EGCg. EGCg quenched the fluorescence of HSA and HSA-prodan. The difference of intensity (△em) between HSA and HSA-prodan (green arrow) indicates the energy transfer.

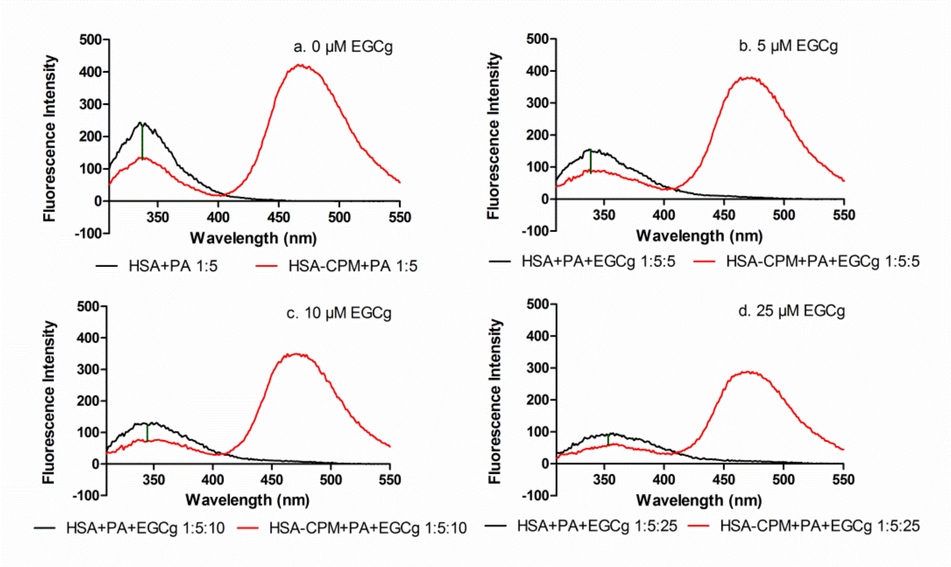

Figure S2. Fluorescence emission spectra for HSA (1 μM)-PA (5 μM) and HSA-CPM (1 μM)-PA (5 μM) with 0 μM (a), 5 μM (b), 10 μM (c), 25 μM (d) EGCg. The difference in intensity at 340 nm (△em) between HSA-PA and HSA-CPM-PA (green line) indicates the energy transfer.

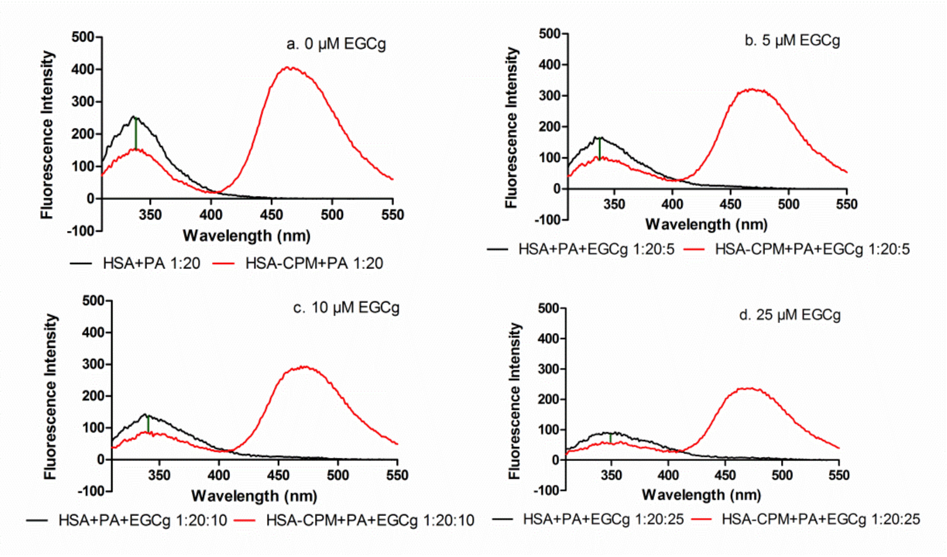

Figure S3. Fluorescence emission spectra for HSA (1 μM)-PA (20 μM) and HSA-CPM (1 μM)-PA (20 μM) with 0 μM (a), 5 μM (b), 10 μM (c), 25 μM (d) EGCg. The difference in intensity at 340 nm ($\triangle_{em}$) between HSA-PA and HSA-CPM-PA (green line) indicates the energy transfer.

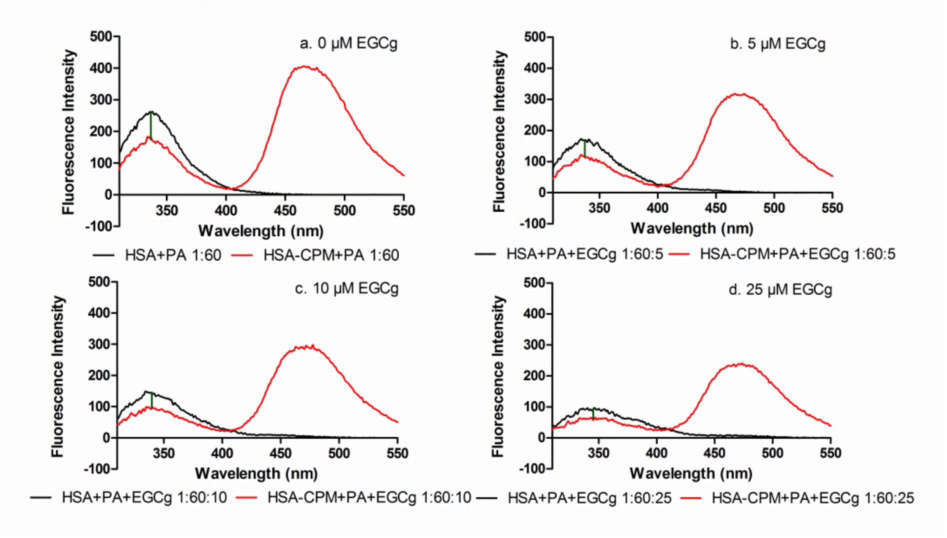

Figure S4. Fluorescence emission spectra for HSA (1 μM)-PA (60 μM) and HSA-CPM (1 μM)-PA (60 μM) with 0 μM (a), 5 μM (b), 10 μM (c), 25 μM (d) EGCg. The difference in intensity at 340 nm ($\triangle_{em}$) between HSA-PA and HSA-CPM-PA (green line) indicates the energy transfer.

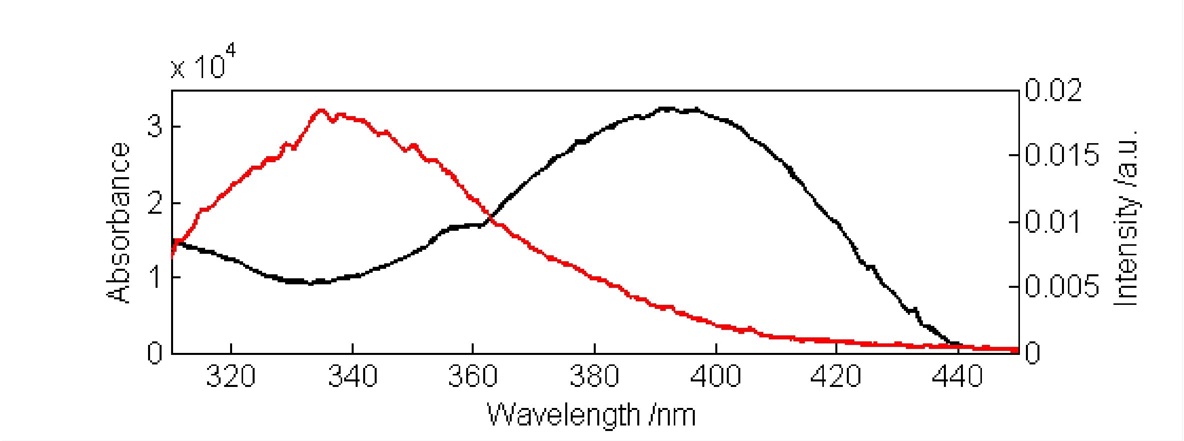

Figure S5. Overlap of the donor emission spectrum (Trp-214) and the acceptor absorption spectrum (HSA-CPM). The J value was calculated based on the overlap of the emission spectrum donor HSA excited at 295 nm (red) and the absorption spectrum of acceptor HSA-CPM (black).

Figure S6. CD spectra of 5 μM HSA and 5 μM HSA in the presence of 25 μM PA, and 0-125 μM EGCg. Samples were dissolved in 20 mM phosphate buffer pH 7.

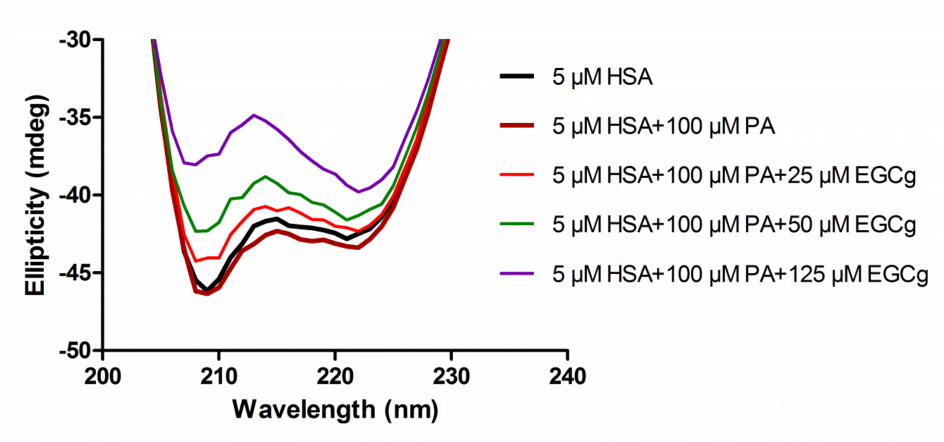

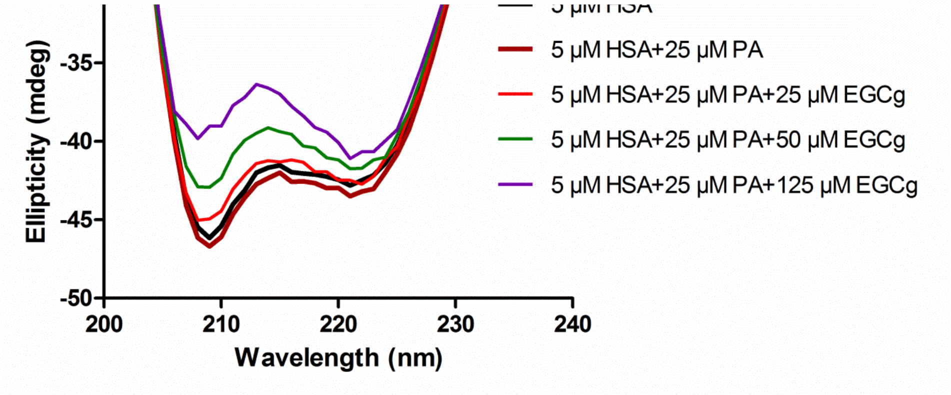

Figure S7. CD spectra of 5 μM HSA and 5 μM HSA in the presence of 100 μM PA, and 0-125 μM EGCg. Samples were dissolved in 20 mM phosphate buffer pH 7.

Figure S8. CD spectra of 5 μM HSA and 5 μM HSA in the presence of 300 μM PA, and 0-125 μM EGCg. Samples were dissolved in 20 mM phosphate buffer pH 7.

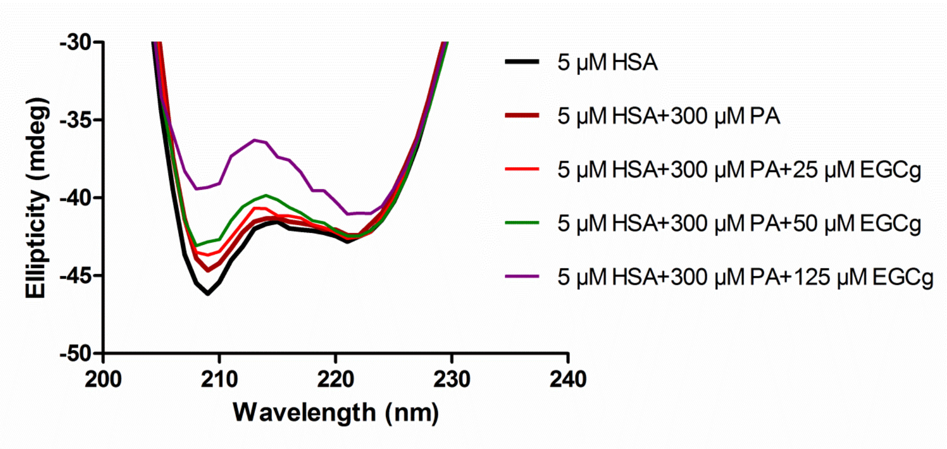

Table S1. J, R0, and R values for HSA, HSA-PA, and HSA-PA-EGCg for three replicates.

HSA:PA:EGCg	J (E14)	R0 (Å)	R (Å)
1:0:0	2.31±0.39	28.09±0.82	30.19±1.51
1:0:5	2.77±0.27	28.98±0.49	32.13±1.01
1:0:10	2.85±0.22	29.12±0.39	32.32±1.09
1:0:25	3.14±0.17	29.59±0.28	33.03±0.8
1:5:0	1.94±0.53	27.22±1.34	29.73±1.63
1:5:5	2.17±0.43	27.78±0.97	31.75±1.17
1:5:10	2.22±0.35	27.92±0.77	31.91±1.35
1:5:25	2.44±0.28	28.37±0.53	34.22±1.27
1:20:0	2.06±0.64	27.45±1.58	32.34±1.90
1:20:5	2.22±0.61	27.84±1.39	33.04±2.11
1:20:10	2.28±0.55	27.98±1.21	33.60±1.61
1:20:25	2.55±0.41	28.56±0.78	36.18±1.92
1:60:0	2.65±0.33	28.75±0.60	35.60±1.12
1:60:5	2.77±0.34	28.98±0.60	36.08±1.51
1:60:10	2.83±0.38	29.08±0.67	36.59±1.15
1:60:25	3.11±0.48	29.52±0.79	37.83±1.13

Table S2. The change in distance (Å) between Trp-214 and CPM induced by addition of EGCg and/or palmitic acid. Within each column of data, the lower case letters indicate the statistical differences for the change in inter-domain distance for a constant palmitic concentration and different EGCg concentrations. Within each row, the upper case letters indicate the statistical differences for the change in inter-domain distance for a constant EGCg concentration and different palmitic acid concentrations. All samples contained 1 μM HSA.

	Palmitic acid concentration			
	0 μM	5 μM	20 μM	60 μM
0 μM EGCg	$0.00^{a,A}$	$-0.46\pm0.12^{a,B}$	$2.15\pm0.46^{a,C}$	$5.40\pm0.46^{a,D}$
5 μM EGCg	$1.94\pm0.51^{b,AB}$	$1.56\pm0.38^{b,A}$	$2.85\pm0.65^{ab,B}$	$5.89\pm0.06^{a,C}$
10 μM EGCg	$2.12\pm0.42^{b,A}$	$1.72\pm0.27^{b,A}$	$3.41\pm0.49^{c,B}$	$6.40\pm0.37^{b,C}$
25 μM EGCg	$2.84\pm0.72^{b,A}$	$4.03\pm0.41^{c,B}$	$5.98\pm0.64^{d,C}$	$7.64\pm0.39^{c,D}$

Table S3. The α-helical content (%) of HSA with various amounts of EGCg and/or palmitic acid. Within each column of data, the lower case letters indicate the statistical differences for % α-helix for a constant palmitic acid concentration and different EGCg concentrations. Within each row, the upper case letters indicate the statistical differences for the % α-helix for a constant EGCg concentration and different palmitic acid concentrations. All samples contained 5 μM HSA.

	Palmitic acid concentration			
	0 μM	25 μM	100 μM	300 μM
0 μM EGCg	$41.46\pm1.47^{a,A}$	$41.30\pm1.04^{a,A}$	$41.08\pm0.39^{a,A}$	$38.98\pm1.23^{a,B}$
25 μM EGCg	$40.19\pm1.17^{ab,A}$	$37.89\pm1.24^{b,A}$	$37.99\pm0.31^{b,A}$	$37.23\pm0.22^{ab,B}$
50 μM EGCg	$38.34\pm1.08^{bc,A}$	$36.17\pm0.52^{b,B}$	$36.47\pm0.43^{c,B}$	$36.44\pm0.52^{b,B}$
125 μM EGCg	$34.23\pm0.90^{d,A}$	$33.20\pm0.19^{c,AB}$	$31.49\pm1.20^{d,B}$	$32.77\pm0.44^{c,B}$
